# The Impact of Children’s Pre-Adoptive Traumatic Experiences on Parents

**DOI:** 10.3389/fpsyt.2019.00866

**Published:** 2019-12-18

**Authors:** Sara Skandrani, Aurélie Harf, Mayssa’ El Husseini

**Affiliations:** ^1^Clipsyd EA4430, Université Paris Nanterre, Nanterre, France; ^2^Maison des Adolescents - Hôpital Cochin, Paris, France; ^3^INSERM U1016 Institut Cochin, Paris, France; ^4^CHSSC EA 4289, University of Picardie Jules Verne, Amiens, France

**Keywords:** pre-adoptive trauma, adoptive parents, reflective function, traumatic impact, adoptive children, otherness

## Abstract

For the last decade, children are adopted increasingly at an older age. Their pre-adoptive past can bare traumatic experiences consequent to abandonment, violence, or deprivation in birth family or orphanage. The objective of this study is to explore the impact of the child’s traumatic past on parental representations and subsequent parent-child interactions. The study includes 41 French parents who adopted one or more children internationally. Each parent participated to a semi-structured interview, focused on the choice of country, the trip to the child’s native country, the first interactions with the child, the knowledge of the child’s pre-adoptive history. The interviews were analyzed according to a qualitative phenomenological method, the Interpretative Phenomenological Analysis. Five themes emerged from this analysis: absence of affects in the narrative; denial of the significance of the child’s traumatic experiences; perceptions of the uncanny concerning the child; parental worry about traumatic repetition for the child; specific structure of the narrative. These extracted themes reveal a low parental reflective function when the child’s past is discussed. They highlight the impact of the child’s traumatic past on parents. Exploring the impact of the child’s traumatic experiences on adoptive parents enables professionals involved in adoption to provide an early support to these families and to do preventive work at the level of parental representations and family interactions.

## Introduction

Before adoption, international adoptees often experience insufficient medical care, malnutrition, maternal separation, neglect, and abuse as well in orphanages as in their family of origin. This risk to live traumatic experiences is even higher, when children are adopted at an older age. Adoption-research is interested in the impact of these adverse pre-adoption experiences on the children’s social and emotional development (1, 2), but also on their family relations and the filiation process.

Adverse pre-adoption experiences increase the risk of post-adoption psychosocial maladjustment, especially with externalizing problems (3–6). But a 50-year follow-up research—The British Chinese Adoption Study—gives as an example of good outcomes in psychological and social adjustment, despite early years of adversity. Scores on mental health assessments were equivalent to the non-adopted, age-matched comparison group (7). However, an important theme, that emerged from the analysis of the children’s adjustment, is trauma (8).

Postadoption experiences, such as quality of parent-child relationship mediate these preadoption experiences to produce different outcomes (9). Even when exposed to preadoptive stressors—such as prenatal substance exposure, child maltreatment, and out-of-home placements— the family sense of coherence has a significant impact on adoptees’ psychosocial adjustment (10). The impact of adverse pre-adoption experiences on the adoptees’ mental health—their psychological distress, externalizing problems, and even academic achievement—is mediated by the quality of parent-child relationship (11, 12) and the parental stress (13).

The post adoptive family relationships thus represent a protective factor on the adoptee’s emotional and social development. It is thus important to understand the impact of these adverse pre-adoption experiences on the parents, the challenges they face as well as their parental abilities (14–16). Using the Parent Development Interview (17) with adoptive parents, Steele et al., (18) showed that parents of late placed adopted children and children with multiple placements prior to adoption, experience higher levels of anger and hostility toward their children, experience a greater need for support, and report higher levels of child aggression, child rejection, child controllingness, and overfriendliness. Cairns (19) explores the phenomenon of ‘secondary traumatic stress’ in which caring for their children who have been traumatized has a traumatizing effect on the adoptive parents. Wilburg (20) shows how the traumatic experiences of adoptees impact their mothers’ mental health, as they suffer from one or a combination of chronic stress, depression, or anxiety after their adoptions. The confrontation with the child’s trauma disrupts the filiation process (21) and is associated with more placement moves, adoption disruption, and inconsistent parental commitment (22). These disruptive relations in persons with traumatic experiences result from defensive mechanisms deployed. Denial is a common defense mechanism observed in traumatic experiences. It serves the adaptive capacities of the psyche to contain traumatic experiences that remain cleaved away from the thinking capacity (23).

This qualitative study focuses on parents’ experiences of their child’s pre-adoptive, and possibly traumatic, experiences. Adopting parents are indeed confronted with their children’s pre-adoptive experiences through the narrative given by the orphanage or by the institutions in charge of the adoption, or through the children’s accounts when they are adopted at a later age. These pre-adoptive experiences can be traumatic for the children as a result of separation from their birth mother or family, life conditions in the biological family or at the orphanage, somatic illnesses or accidents, or ill treatment, neglect, or abuse. What impact does this confrontation with their children’s pre-adoptive experiences have on adopting parents, and more precisely on the development of a filial link and parental representations of their child? This issue was addressed in the present study using semi-directive interviews with parents who had adopted one or several children from abroad.

## Materials and Methods

### Participants

The participants of the research adopted at least one child from a country other than France. No other inclusion criteria were defined. The sample consisted of 41 adoptive parents who volunteered to participate in the study, 31 mothers and 10 fathers. This gender difference is due to a higher acceptance for participation among mothers. The sample size was determined by data saturation: when the in-depth analysis of the interviews no longer produced the emergence of new analytic themes, we stopped the inclusion of new participants.

**Figure 1:**
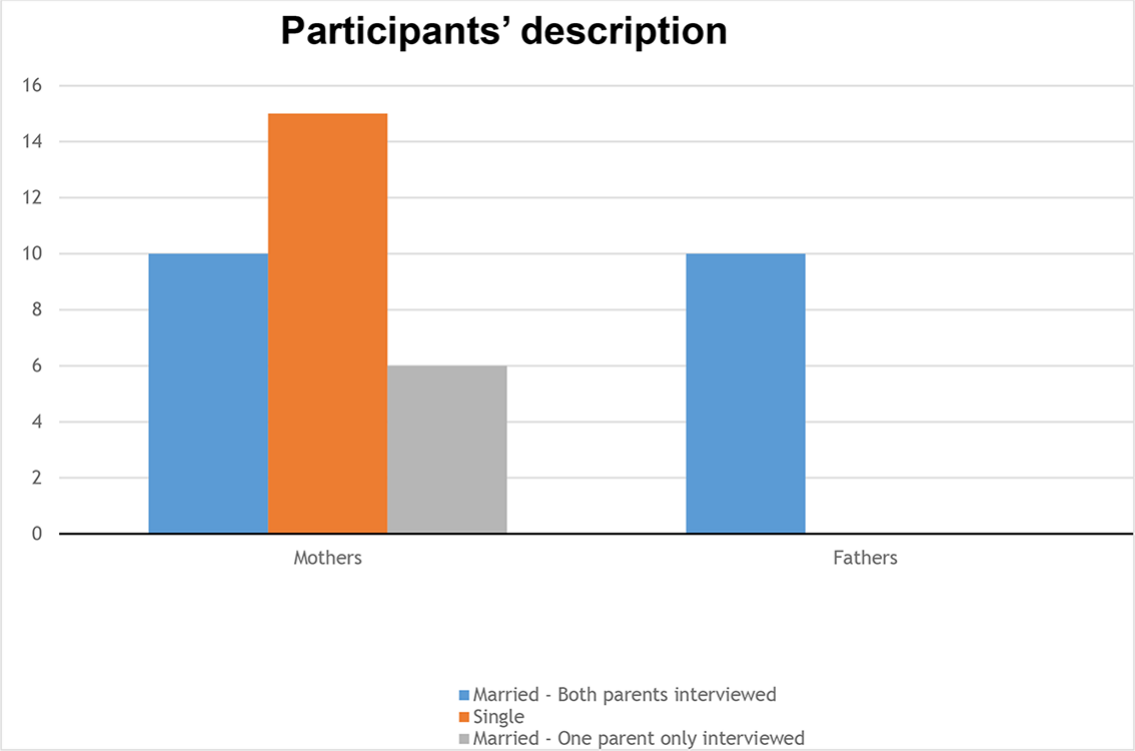
Particpant’s description.

Overall, 26 parents were married and 15 had adopted as single parents. When children had been adopted by couples, we asked both parents to participate in the research. Interviews were performed separately for each parent. The 41 parents in this study included 10 couples in which both parents (father and mother) participated, that is, 20 parents. The remaining 21 parents were mothers: 15 adopted as single mothers, while 6 adopted with their husbands, who did not participate in the study (see **Figure 1**)

**Figure 2:**
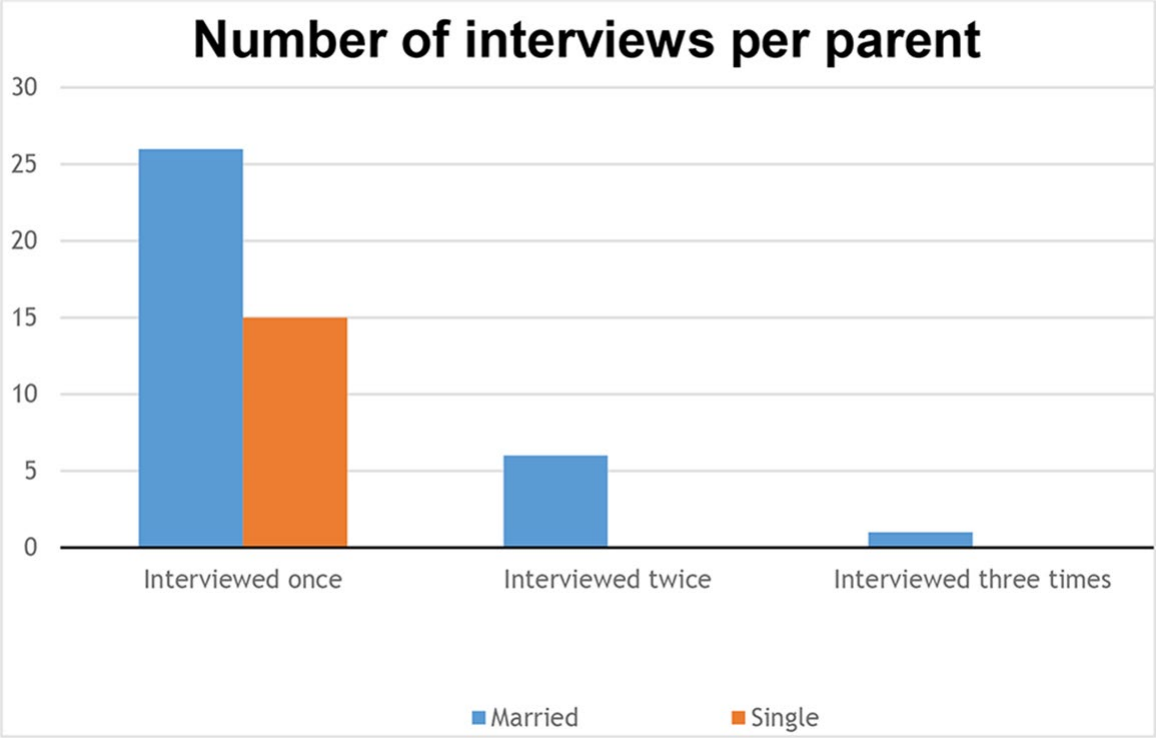
Number of interviews per parent.

Six parents had two internationally adopted children and one mother had three. They were interviewed respectively twice or three times (one interview for each child). We conducted a total of 49 interviews (see **Figure 2**).

Parents’ ages at the time of their children’s adoptions ranged from 28 to 49 years and at the time of the interview from 31 to 60 years.

Parents lived in urban areas of France. Most (n = 38) were college-educated professionals. They were recruited through adoption associations and connections between adoptive parents.

**Figure 3:**
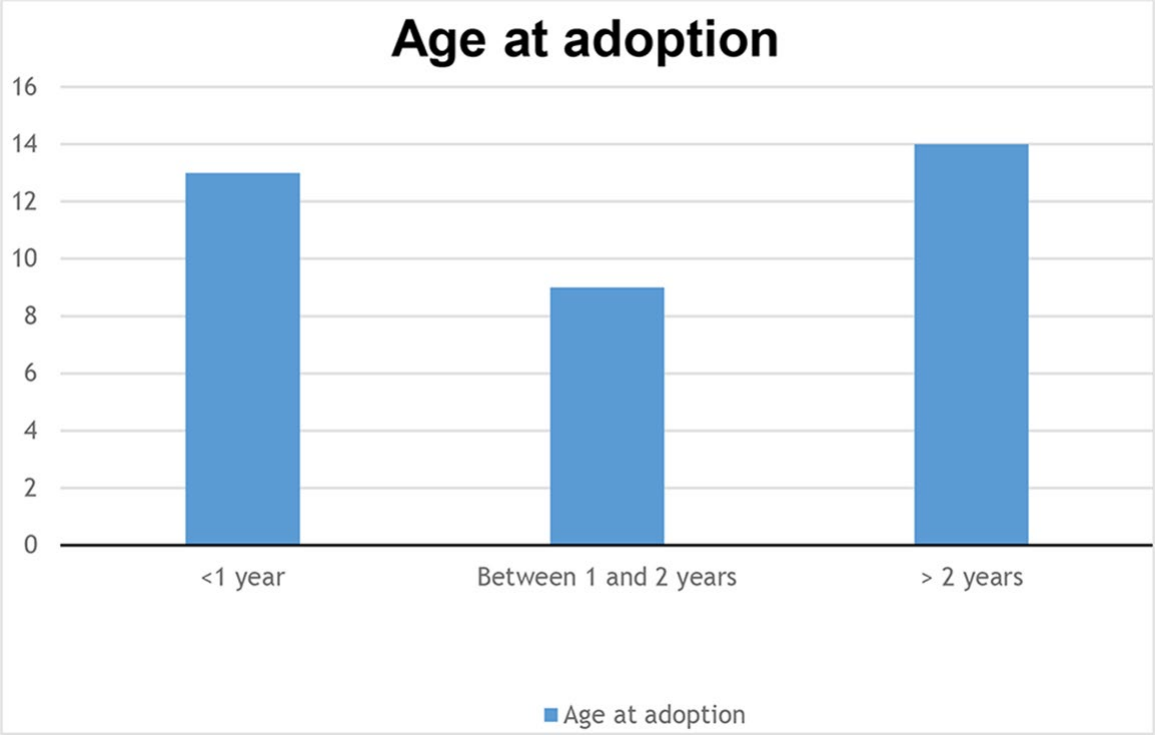
Age at adoption.

These 41 parents had adopted a total of 36 children: 19 were girls and 17 boys. At the time of their adoption, their ages ranged from 2 weeks to 7 years. Thirteen children were younger than 1 year at the moment of their adoption, 9 were between 1 and 2 years old at adoption and 14 were older than 2 years. They were adopted in the following countries: Algeria, Armenia, Bulgaria, Brazil, Cambodia, Central African Republic, China, Colombia, Ethiopia, Guatemala, Haiti, India, Lithuania, Madagascar, Mali, Poland, Romania, Russia, Thailand, and Vietnam. At the time of the interview, the children’s ages ranged from 15 months to 17 years (see **Figure 3**).

The sample was thus diversified in terms of age, life stage (i.e. families with young children or adolescents), and family structure (single parents and married couples). Following a qualitative research sampling technique (24), we adopted a purposive sampling, as we selected subjects who were typical for the population of interest.

### Data Collection Procedure

We conducted semi-structured interviews. The interview guide was developed after a review of the international adoption literature. Different topics were covered, including the choice of country, the trip to the child’s native country, the first interactions with the child, the knowledge of the child’s pre-adoptive history.

The interviews were conducted at the researcher’s office or the parent’s home, at their convenience. Their average length was 1 hour, depending on the participants narratives. With their permission, the interview was audio-taped and later transcribed. The research material was then analyzed by two different researchers (AH, SS), each of them trained in the fields of international adoption and qualitative research methods.

Questions were designed to obtain specific information while remaining flexible so that the interviewees could tell their stories. Open-ended questions allowed participants to interpret the meaning of the question and respond according to their personal feelings. Interviewers used prompts and probes as needed to enrich the discussion. We chose to collect data through semi-structured interviews because this method combines an approximate standardization of questions with the opportunity for subjects and interviewers to expand their answers when appropriate. The interviewing process produced deep and broad data that focused on the research question: how subjects described their experience of their first meetings with the child they were adopting.

### Data Analysis

The interview data was analyzed according to the Interpretative Phenomenological Analysis method (IPA) (25). This qualitative research method permits the exploration of the participants personal experiences and unique representations of their child’s pre-adoptive history, through a detailed examination of the participants’ personal perceptions and lived experiences. We thus conducted an in-depth qualitative analysis. Through an iterative inductive process, we proceeded to a detailed case-by-case study of each interview transcript. We began with several close detailed readings of each interview to provide a holistic perspective, noting points of interest and significance. Through a step-by-step analysis, analytic themes emerged, which were described as well as their interconnections, while preserving a link back to the original interview material. This process produced a coherent ordered table of the themes. This data analysis procedure was inductive, as the analysis of the results of international literature on this specific subject was performed in the aftermath.

To assist this analysis process, we employed a qualitative analysis data software: QSR NVivo for data management, topic extraction from, and thematic recodes of individual interview transcripts.

### Validity

We compared the researchers codings, to insure the validity of our qualitative research. The three researchers involved in this study are specialized clinicians in family therapy, transcultural psychiatry, trauma, and international adoption.

Two trained researchers (AH and SS) independently coded and interpreted the parent’s interviews. The emerging codes were repeatedly discussed with another research team member (MH) who had read the transcripts. These discussions permitted to identify additional themes in the data, that might not yet have been described by the codes. It enabled us to add or modify the coding in order to increase the consistency and coherence of the analysis. It was thus ensured that the themes were accurately identified and reflected the data. Through this process, systematic differences, due to variations in interpretation, were eliminated. Validity was also enhanced by distinguishing clearly between the respondents’ discourse and our interpretation (25).

### Ethics Statement

Parents were fully informed of the voluntary nature and the goals of the study. Written informed consent was obtained from all parents included in the study before the interview. Participants were informed that all responses would be confidential, that the transcripts would have no identifying information, and that they would be free to withdraw at any time. All identifying information was removed from the transcripts, and participant anonymity further ensured by disguising or withholding of descriptive data. Additionally, written informed consent was obtained from the participants for the publication of their indirectly identifiable data.

## Results

In half of the interviews conducted (n = 24), parents reported traumatic pre-adoptive experiences for their child: sudden loss of their biological mother, repeated experiences of neglect or abandonment in the biological family, emotional deprivation at the orphanage, major health problems, or serious accidents or injuries.

An interpretative and phenomenological analysis determined five themes in the interviews with the parents. Three of these themes can be sub-divided into several sub-themes (see **Table 1**).

**Table 1:** Participants’ description.

Themes extracted from the analysis	Subthemes	Number of parents interviews’
Absence of affects in the narrative		12
Denial of the significance of child’s traumatic experiences	*Denial of the child’s pre- adoptive past Denial of the child’s health problems and suffering* *Difficulty, inability to talk about the child’s histor*y	14
Perceptions of the uncanny concerning the child;	*Feelings of rejection in relation to the child* *Cultural devaluatio*n	13
Parental worry about traumatic repetition for the child		5
Specific structure of the narrative.	*Disorganisation, contradictions and fragmentation of the narrative Incoherence of the narrative* *The presence of silences* *The pervasion of the narrative by a single theme* *The interviewee’s absorption in scenes of the past* *Physical symptoms* *The presence of undefined (stop-gap) words*.	18

### Absence of Affects in the Narrative

The first theme identified concerned the absence of affects. Twelve parents reported pre-adoptive traumatic experiences by their child, however they did not express any emotions—whether in relation to the child’s experience, or to their own.

V. lost her biological mother very suddenly. She had been living with her on the streets when one day, she got lost in an unknown city. She thus found herself in an orphanage when she was four years old. When V.’s adoptive mother mentioned her daughter’s life in the orphanage, she described it as a “prison.” According to her, the child’s difficulty lay in adapting to this imprisonment after her “nomadic” life, without any mention of the pain, the dismay, or the despair her daughter might have experienced because of the traumatic loss of her mother.

“She was very unhappy at the orphanage and this could very well be explained considering that as she had led a nomadic life, and for a child of her age this kind of life has in a way some advantages.”

A.’s biological mother died suddenly when she was one year old, which led her to be placed in an orphanage and adopted six months later. A long time after her arrival in her adoptive family, she asked where her biological mother was; her adoptive mother reported this without making any reference to her child’s despair, having been faced with the incomprehensible loss of her biological mother:

“I think she’s just a little girl, she was taken away from her mother and there’re a lot of things she didn’t understand. I think that in her mind, she was going to go back to Ethiopia. She didn’t understand anything. So it was a little complicated for her. Well, it is still a little bit difficult.”

B.’s adoptive mother talked about her daughter’s health problems at the orphanage, without mentioning the psychological suffering that probably occurred alongside the physical pain experienced by this 5 month-old baby.

“Well, when I think about it now, at the time she arrived here, because when she arrived, she had scabies, it caused her discomfort and she kept scratching herself. She had a massive skin reaction to her anti-scabies treatment. One night she had a rash all over as if she’d been burnt all over her body, she was screaming. So I think that physically she had a lot of discomfort for a large part of her life, because she probably had scabies during the whole time at the orphanage.”

These parents reported pre-adoptive traumatic experiences without mentioning any internal experiences, or minimizing their child’s affects or emotions. They gave an account of this painful pre-adoptive life without expressing any affects themselves and without referring to the impact of the history on their feelings as parents.

### Denial of the Significance of the Child’s Traumatic Experiences

Denial of the significance of traumatic experiences in the children’s pre-adoptive histories was found in the interviews of 14 parents. Parents reported different traumatic experiences—intra-familial violence and serious parental neglect in the biological family, sudden and incomprehensible separation from the biological mother, accidents, and major life-threatening health problems. They however denied the traumatic significance of these events and their impact on their children’s emotional experiences and their later psycho-affective development. This theme can be divided into three sub-themes: denial of the child’s pre-adoptive past, denial of the child’s health problems and suffering, difficulties, or even inability to talk about the child’s history.

#### Denial of the Child’s Pre-Adoptive Past

A long time after the adoption, A. continued to ask for her biological mother—“Naté” in Swahili—who had died suddenly when A. was one year old. After having mentioned this aspect of his daughter’s lifeline in factual terms, her father did not talk about it again during the interview. He focused however on relationship difficulties his daughter had regarding the adoption itself—thus shutting off her previous life. His answer to the question about difficult times experienced by his daughter in her life was hesitant, it was interrupted with silences and changes of subject. It showed his reluctance to talk about this subject and highlighted the father’s inner conflict to neutralize any significant traumatic impact on his daughter.

“(Silence)… The adoption. The trip. Well, the adoption… (pause), the move here, her friends. At the same time, she’s quite curious. She’s quite … I would say that it’s really about the adoption.”

When C.’s father was interviewed about events that led his daughter to be adopted, he exclusively mentioned her own wish to be adopted.

“Well, what made her decide, well, first it was … in fact they asked her if she wanted to, and so, I think she saw, er, psychiatrists or psychologists to see if she wanted to, if she really wished (…) to be adopted (…) So she was completely aware … so.”

Reasons which let C. to be removed from her biological family, to be placed in an orphanage and to be adopted, such as experiences of ill treatment by her biological family, seemed to be repressed. Instead, his narrative placed C. as the instigator of her own adoption, thus denying the trauma endured as a subconscious way to be both protected from the disrupting traumatic impact.

H.’s adoptive mother on the other hand considered that her daughter’s abandonment and adoption were her own initiative and desire. When questioned on the reasons why H. was abandoned, she gave the following answer:

“Yes, because once adopted … It was me who adopted her, so…”

She talked only about her own family and transgenerational history to give meaning to her daughter’s adoption, without mentioning the child’s painful pre-adoptive past in any way. Focusing on her own lifeline is indicative of the denial process which targets the traumatic impact of the daughter’s past, which was suppressed from the narrative.

Denying the importance of a child’s pre-adoptive past is thus a way of denying the significance of the traumatic experience itself. This denial was again unfolded by the obliteration of any memories relating to the trauma—in this case extreme parental neglect leading to the removal of the child from parental authority.

“I think that his … his memories, they don’t go beyond … the orphanage, so his first memories are linked to the orphanage.” (L.’s mother)

#### Denial of the Child’s Health Problems and Suffering

Denial of the significance of the child’s traumatic experiences was linked to a sub-theme concerning the child’s health problems and suffering.

When she was a child, V. had two serious illnesses, probably linked to her very precarious life conditions in the streets with her biological mother, whom she later lost. When she was adopted, doctors realized that she had already developed pneumonia in the past, with somatic after-effects, as well as hepatitis B.

“We found out that she had also contracted hepatitis B, but apparently she got rid of it, I mean, she’s developed antibodies. But we don’t know how she caught it. Anyway, she didn’t need to be vaccinated and that’s a very good thing.”

Without referring to her daughter’s subjective experience when she was ill, her adoptive mother only underlined the positive aspect: V. did not need to be vaccinated. The deployment of defense mechanisms as minimization or even reversal of meaning—”that’s a very good thing”—of her daughter’s health problems, can be understood as an attempt to deny their impact on the child and the meaning they carry: the evidence and consequence of her earlier hostile, difficult life conditions. The adoptive mother concluded with a statement that went against what she had reported earlier: “she hasn’t had any serious problems.”

Similar statements were made by F.’s mother when she was asked about her daughter’s potential health problems.

“No … Well F. is a little girl who was burnt, she was seriously burnt. She fell into boiling water when she was little, so she … she was in hospital a long time, she had skin grafts. There are burns on her body … I can’t remember. I think it’s 60% of her body, so there we are. Otherwise, she’s a child with no major health problems.”

The traumatic experience of having fallen “into boiling water” when she was not even two, which seriously burned a large part of her body, requiring many skin graft operations, was totally minimized by the mother—“no major health problems.” This could be interpreted as an attempt to annul the unbearable trauma experienced by her daughter, whose body was an ever-present reminder.

#### Difficulty, Inability to Talk About the Child’s History

Certain parents only mentioned their child’s pre-adoptive past with a repression of affects or comments, despite the fact that this past was really frightening, even shattering.

“So I don’t know, it’s probably someone who attacked her, well, with a machete or goodness knows what, she had a hole here, behind her head, a hole, not in her skull but on her hair’ (I.’s mother)

This extremely violent experience suddenly emerged from this mother’s account, with its potentially dazing impact, but she did not go back to the subject and was unable to speak about it any further.

Other parents did not wish to talk about their child’s pre-adoptive traumatic past. F.’s father talked about his daughter’s pre-adoptive past in these terms:

“The Lithuanian files had been well compiled so we were informed of events with her family and we were aware of her health problems, in particular because she had been so seriously burnt.”

When the interviewer repeated the question, he refused to talk any further: “No, I think that answers your questionnaire.”

This was the case with B.’s mother who wished to leave her daughter “the primacy of her history.” However, she felt “stressed” by the professionals who were questioning her about the past. They were worried for B., especially on account of alarming diagnoses they had made on her arrival in her adoptive family. It was thus through the professionals’ reaction to her daughter’s history that the mother felt alarmed. Fears linked to her daughter’s past were kept silent. But that past was experienced and recognized as such, through the eyes of professionals on her daughter.

B.’s father expressed his refusal to talk about his daughter’s pre-adoptive past even more clearly.

“She was er, (silence), so (silence), the circumstances in which she was abandoned, well, considered as abandoned (silence) and as for the remaining circumstances, er, we don’t tell anyone.”

Refusing to talk about the child’s painful or traumatic past can be seen as a painful attempt to occult its existence and impact.

### Perceptions of the Uncanny Concerning the Child

Perceptions of “the uncanny” emerged from many interviews (n = 13) on the part of the parents toward their child. Furthermore, this emerging theme could be linked to two sub-themes: rejection and resorting cultural devaluation.

When A.’s mother mentioned the anxiety and distress expressed by her daughter, she underlined their “sordid” nature. This term reflects the uncanny element felt by the mother in this context, which made physical contact between the two difficult in moments of affection.

“Even now, when she wants a cuddle, it’s always extremely clumsy. As for me, in front of her, I’m also clumsy. Contact is difficult … In fact, when she needs physical contact, I have to reason myself and tell myself: relax, let her come to you.”

D. suffered from severe malnutrition in infancy. According to his father, he was only “saved” once he got to the orphanage. The relationship with food that he later developed was seen as the consequence by his adoptive father, which greatly worried him.

“The minute it’s in his plate, he’s like an animal, really, a wounded animal, and that’s worrying, for me, it’s something that worries me, his impulsive side, but it’s not impulsive, it’s irrational, and I’m worried about it, it scares me, and then, with adolescence, how far can it go at that age? (…) It’s almost like bestiality, it’s really … very basic instinct.”

The use of this term “bestiality” perfectly illustrates the perception of the uncanny experienced by the father concerning his son. Because of his malnutrition experience, D. was seen as a « wounded animal » whenever he had food in front of him, which removed him from the human community. Parental expressions comparing their children to animals or things or mysterious objects were found in many of the interviews.

“He’s like a wild cat who has set up strategies … of defense. I mean, it doesn’t take much for him to show his claws” (T.’s mother)

“F.… er … when we first met her, she was … She was like a pot of flowers (…) I mean, she was a little girl who did not take part in anything and was an outsider to everything.” (F.’s mother)

“But with the international adoption scheme, when I brought my daughter back with me, she was a sort of UFO” (V.’s mother)

This perception of the uncanny toward their children was seen as worrying.

“It worries me” (C.’s father)

In accounts from other parents, it was the country of origin and its culture that crystalized this perception of the uncanny: a “mysterious” and unfamiliar country (I.’s mother), an “overdosing” country (T.’s mother) and a world like a “concentration camp” (M.’s mother).

#### Feelings of Rejection in Relation to the Child

This perception of the uncanny in the child was not only felt as worrying, it could even lead to the child’s rejection by the parents.

“I think that since he’s been here, the word that sums it up is it’s really difficult. Difficult with the family, difficult in his relationships with his sisters, difficult with us, difficult at school and difficult in his relationships with his friends, so there we are (…) it’s true that he shows up the bad side of adoption (…) Well, we are sick and tired, so it’s true, sometimes we say things that go beyond our thoughts, but we’re really fed up” (R.’s mother)

“I actually hate him! I can tell you this and shock you, so what” (T.’s mother)

Parents also experienced rejection by their children.

“We are not very close affectively (…) I don’t know whether I’m essential in her life. I don’t feel she needs me.” (I.’s father)

“She really rejected me at first. Really, really, really rejected me (…). Well, I mean, she was only very little. But it was really painful” (A.’s mother)

A.’s mother was not able to link this reaction to her daughter’s recent pre-adoptive experiences: her biological mother’s sudden, unexplained death, and the fact that she asked after her for a long time. The adoptive mother only talked about her own internal experience and the pain she felt at this rejection. In this context, she mentioned the need “to protect herself” from this painful feeling.

#### Cultural Devaluation

A second sub-theme linked to perceptions of the uncanny discussed previously concerns what El Husseini et al. (26) referred to in their studies as cultural devaluation.

T.’s mother linked her son’s traumatic past—an infant who had repeatedly been left alone for several days by his alcoholic parents—to what she called the “Slavic culture.”

“There’s a lot of alcohol and violence over there … So kind words, things like that … It’s not in their culture. There it is.”

This child’s experience of extreme distress, abandoned, alone and without food, was confused with his cultural origins. Her son’s unbearable experience was attributed to his culture of origin—the “Slavic culture,” violent, unloving, alcoholic.

The impact of the trauma on the mother pervaded her whole implicit perceptions of the country of birth and the child’s attachment to his origins, familial as well as cultural. In this context, the mother expressed her incomprehension, even her non-recognition of the need expressed by her son to have links with his biological mother, and her surprise at her son’s difficulty in “losing” his mother tongue.

Before she was five years old, F. underwent several skin graft operations following her serious, extensive burns, which meant that she had to be isolated a lot. The mother’s confrontation with this pre-adoptive history and with her daughter’s badly marked, mutilated body following these traumatic experiences established links with the culture of her daughter’s country of birth.

“There’s the individual history, there’s also the history of … that country. So we went to orphanages (…) well, I saw some real harridans, some Belarusians that were there, and they were like prison wardens (…). I tend to put everything in one bag, but it’s true, there are things that can’t be forgotten. We can’t forget (…) it’s not just her personal history, there’s also this country’s history, where people talk loudly, and hit out easily. I think that’s part of it.”

What this girl had to live through physically and psychologically was, because of its violence, unthinkable for the woman who became her mother. The unbearable nature of her daughter’s traumatic experience undermined the mother’s ability to elaborate—“(…) but it’s true, there are things that can’t be forgotten. We can’t forget.” Being unable to give meaning to the situation, she looked for it in the culture of origin where these traumatic events took place—“the country’s history,” full of “prison wardens,” noisy and violent.

### Parental Worry About Traumatic Repetition for the Child

In a few parental interviews (n = 5), parental worries were found with regard to the potential recurrence of the trauma in their children. The significance of certain current experiences was interpreted in the light of the child’s pre-adoptive experience.

C.’s father was very worried during prolonged separations from his daughter, leading him to set up protective strategies in case of emergency: “I was more worried in the beginning (…). But as parents, we get more worried when we send her to a summer camp, it reminds her of the orphanage, especially in Poland. So we were anxious for everything to go smoothly. At least (…), at least, my wife wasn’t far away. She was in Poland, staying with her brother, about 100 km away, she could go and get her. At least we had this safety net.”

L.’s father associated his son’s recent racketing experiences at school with the feeling of painful abandonment he had experienced before his adoption: “He knows he was abandoned, I think he suffered at not having a mother at the orphanage, because the other children, three quarters of the other children had been placed there, but the parents in fact came at the weekend or once a month, I think that he suffered a lot, L., the youngest boy, not having anybody.”

T.’s mother understood her son’s violent behavior as a recurrence of the “violent past of the family.”

N.’s mother interpreted any feeling of rejection experienced by her son in his relationships with others as an endless repetition of his biological mother’s initial abandonment. When she felt that her son was “lost” or distressed, she would “(take out) his little hat,” knitted by his biological mother or a photo of her. Similarly, she associated his “somatic symptoms”—stomach ache, pain in his feet—with the malnutrition he had suffered during the first three weeks of his life: “yes, he does have somatic symptoms (…) but it could well be linked to undernourishment, couldn’t it? When I had him, he was undernourished.”

F.’s mother wondered about her daughter’s future pregnancies because of the lack of love and the many rejections she had experienced on the part of her biological mother and father, but also because of how she saw her body, bearing the marks of serious injuries.

### Specific Structure of the Narrative

We explored the structure of the parents’ narrative when they started talking about their child’s pre-adoptive life, what they knew about the abandonment and the child’s personal history before the adoption. An analysis of the narrative structure showed specific and recurrent characteristics with the same narrative format in many of the interviews (n = 18). These specificities are as follows: disorganization, contradictions, and fragmentation of the narrative; incoherence of the narrative; the presence of silences; the pervasion of the narrative by a single theme; the interviewee’s absorption in scenes of the past; physical symptoms; the presence of undefined (stop-gap) words.

#### Disorganization, Contradictions, and Fragmentation of the Narrative

Disorganization and fragmentation of the narrative was found in 12 interviews with the parents. This can be defined by loss of the train of thought, incomplete and interrupted sentences, and contradictory and broken narratives.

“Yes, I think there’s a date she talked about, the day she … She remembers when she arrived at the orphanage. It was traumatic. From what I understood, she arrived there with the police, etc. I think that when she realized she had been abandoned, it’s something that … One thing she’s talked to us about is the day she … Apparently, her mother…” (C.’s father)

“I think so, yes, all these periods when she was alone, in fact, when she was crying. I also think … For the others, we had a few clues written on paper, for her we have nothing … So, because of that, maybe we tend too much to think that it’s been harder for O., even without … well, subconsciously” (O.’s mother)

Sometimes the parents’ accounts were incoherent and difficult to follow.

Concerning the issue of losing his mother tongue, T.’s mother lost the thread of her narrative: she went back to the process of adoption itself, their difficult experiences, her time off work, the return to work, before asking herself ‘Why am I telling you all this? I wanted to fall back on my feet by telling you all this and there we are.”

On many occasions during the interview, F.’s mother forgot the question that had been asked before, carried away by her story and the intensity of the feelings and affects described, particularly when she recounted her daughter’s painful past: “Er … for, er … for, er, F.… what was the question again (laughs)?…”

When V.’s mother was asked to describe her daughter, she lost her train of thought and could not remember what she had said: “So, what did I first tell you? I’ve already forgotten! (…) And what did I say last? I’ve forgotten. Ah, yes, I said a … No, what did I say? I can’t remember what I said.” Alongside, in her narratives there were numerous digressions and details, in a continuous flow of words about her daughter’s problems, which at the same time she minimized.

This disorganization, contradictions, and fragmentation of the narrative was only observed, when the parents talked about their child’s pre-adoptive life.

#### Incoherence in the Narrative

Incoherence in the narrative is defined by a discrepancy between semantic memory and episodic memory (27). It was found in four parents’ accounts.

L.’s father explicitly said that he only had “very little” information concerning the conditions of his son’s abandonment. However, he described them in a very precise way “When he arrived at the orphanage, he was undernourished, he had not been very well looked after … I understand that the mother had been away for a few days. It was the police who took them there in emergency, er … at two and a half years, he wasn’t talking … only a few words.”

C.’s mother started to recount her daughter’s pre-adoptive life by saying that she “knew nothing about her child before she was adopted.” However, she gave a lot of information: “Er … so er … we know that … that she was … brought to the orphanage by the police when she was 5… er … and it’s probably the neighbors who alerted the police, telling them that there were two children in the flat … in the building and … and I think it was winter and in the flat there was no heating, there was no light or there was probably nothing to eat and … two dirty children, alone, who were probably crying and there we are … and … so er … I think it’s the neighbors who called the police and the police brought them to the orphanage to be placed.”

When F. ‘s mother talked about her daughter’s health problems, she reported serious burns requiring many skin graft operations. She then concluded in this way: “Otherwise, she’s a child with … with no major health problems.”

This observed incoherence in the narrative was restricted to the parental accounts of their child’s pre-adoptive experiences. When other aspects of the child’s life, his relations with his parents, the choice of country were addressed, the parental narratives were coherent.

#### The Presence of Silences

The narratives of three parents were introduced and punctuated by many silences.

“She was er, (silence), so (silence), the circumstances in which she was, well, abandoned, well, considered as abandoned (silence)… and as for the remaining circumstances, er, we don’t talk about it to anyone.” (B.’s father)

“(Sigh, silence) well, he doesn’t ask anymore, er, (silence), what questions did he ask? Er, why his mother died; she must have been poor, she was ill, so his grandmother, she looked after him.” (D.’s father)

“(silence)… The adoption. The trip. Well, the adoption. (silence) the move to this place, her friends. At the same time, she’s quite curious. She’s quite … I would say that it’s really about the adoption.” (A.’s father)

#### Pervasion of the Narrative by a Single Theme

Long descriptions with no link to the rest of the narrative were found in several parents’ accounts (n = 5). Their narratives were thus pervaded by a single theme.

When C.’s pre-adoption life was mentioned, her mother deflected from her daughter’s personal experiences and gave an extended presentation of the types of children’s institutions that existed in Poland.

When talking about his daughter’s medical history, in particular scabies, which she had contracted, B.’s father suddenly gave a long account of the geography of Ethiopia:

“‘(…) and er, on top of everything, she had skin complaints, she had scabies so she was constantly scratching herself, anyway there was a lot of discomfort etc. so for us, she was a little girl, so it was, er, beautiful, really, and the country is … beautiful, there’s a lot of contrasts, because it’s like walking in Normandy, there are Normandy temperatures on Ethiopia’s high plateaus, it’s green just like in Normandy, and there’s so much poverty, there’s a lot of contrasts, there are no old people in Ethiopia, there’s a lot of people in the streets, they’re all young, the old people are all dead … Or anyway, they never come out.”

After having described the natural beauty of the country, it was the theme of death that suddenly emerged in his account.

#### Absorption in Scenes of the Past

When they talked about meeting their child at the time of adoption, four parents seemed absorbed in memories from the past.

C.’s father was absorbed in sensory memories “I stayed in the car with her for 1 h, trying to explain: “come with me.” She took hold of my hand, she clawed it with her nails and then “you’re hurting me, but it doesn’t matter.”

M.’s mother reported some shocking images at the time of the adoption:

“We saw him, in big beds with bars this high, what do you call them, you know those white clothes for mad people, you know, the way we strap babies, straitjackets, he was in this kind of garment, you couldn’t see his hands, a large white garment buttoned up on the outside.”

#### Physical Symptoms

Physical symptoms were found in three parents interviewed, showing the intensity of the concomitant psychological processes. C.’s father laughed several times in inappropriate manner during his account, especially when he described painful events experienced by his children and their repercussions. L.’s father coughed a number of times when he talked about the conditions of his son’s abandonment.

#### The Presence of Undefined (Stop-Gap) Words

The use of undefined or stop-gap words, showing a lack of words and representations to describe the child’s experiences, was found in two parents’ accounts.

“Er … When we took him to see the psychologist, this thing he had a problem with, it was hard for him to tell us (…) We didn’t pay that much attention to the fact that he had a funny kind of behavior, that he would wake up at night, he would get irritated, he was … whatever … and then it was the holidays (…). (L.) talks about the adoption … He knows that thingy…” (L.’s father)

When A.’s mother mentioned the anxiety expressed by her daughter, she lacked words: “So, if she, herself, had no more legs, no more thingy, what…? Anyway, there’re only things like that … it’s a bit sordid.”

## Discussion

Five themes emerged from the IPA analysis of the interviews: absence of affects in the narrative; denial of the significance of the child’s traumatic experiences; perceptions of the uncanny concerning the child; parental worry about traumatic repetition for the child; specific structure of the narrative.

These themes reflect the parents’ personal experiences of their children’s pre-adoptive past. They raise the issue of the traumatogenic impact of these traumatic experiences on them and their repercussions on the child’s parental representations. They could be interpreted as the transmission of trauma from the children to their parents (28).

When they mentioned potentially traumatic events experienced by their children before their adoption, half of the parents showed (12/24) a disruption in their affective function—none on the subject of the child and none for their own account. They hardly ever referred to their child’s internal personal experience. Some parents denied the impact of the pre-adoptive past on their child’s affective and emotional experiences, minimizing it or repressing it. When the traumatic events concerned health problems linked to hostile life conditions, these problems tended to be minimized. This denial of a painful, traumatic past could reflect an attempt to cancel, to erase in hindsight, its existence, and the impact on their child. Several studies have shown that the mechanisms of denial and minimization of hostile experiences are criterions found in the narratives of traumatized subjects (29, 30), highlighting the traumatogenic impact of these experiences on the parents.

This absence of affect in the parents’ accounts and the denial of the significance of their child’s traumatic personal experience shows poor reflective function on the part of the parents. The reflective function as defined by Fonagy (31) is the ability of the parents to picture their child’s affective and internal personal experience, to let themselves be affected and to adjust to it, in order to mentalize it. The parents’ mentalization capacity helps the child identify with a meaning to its own disrupting experiences and thus integrate it in their storyline and their world experience. This process alleviates the traumatic impact on the child in its affective development. This internal/psychological dimension, either silenced or canceled, shows parents’ specific difficulties in identifying themselves with their children when their pre-adoptive past is mentioned. This inability to identify themselves—restricted to a given period—can be interpreted as a way to distance themselves from the unbearable, the unthinkable in their child’s traumatic personal experience. On the other hand, when other difficult moments in their child’s life were mentioned, events that took place after their child’s adoption, the parents were quite capable of talking about their child’s and their own personal affective experience, thus showing a good reflective function. This observation supports the understanding that the absence of reference to internal and emotional personal experiences is a form of defensive reaction to the child’s traumatic personal experience. Temporarily, they find themselves incapable of identifying with their children or of putting themselves in their place. This sudden and temporary interruption in their reflective function is a sign of trauma. The results concerning the reflective function seem all the more important, as other research have highlighted its significance as a predictive factor of the child’s outcomes, especially for maltreated adoptees (18, 32). A high reflective function enables the parents to identify with their children, preserving them from a feeling of intense otherness and strangeness.

This adoptee’s position of otherness was precisely found in our results. We postulate that the parental experience of the child’s strangeness and otherness depicts their reaction to the pre-adoption trauma. The terms used by the parents to describe their child expressed a certain dehumanization of their child. The parents’ narratives provides insight into what cannot be thought or elaborated by the parents. The impossibility of representing what the children have lived through could exclude these children from the human community, thus hindering any identification process for the parents. The child takes on an unsurpassable otherness. This otherness has already been described in studies on children’s skin color (33) or on their foreign genetic heritage (34, 35). The present results add another dimension to the child’s otherness, related to the impossible representation of his pre-adoptive experiences. The adoptees holding this trauma through scars on their bodies or through the narratives of their parents could be stranded between two filiations and doomed to wander between affiliations: they have been uprooted from their past world and the rooting process in their present is impeded by the trauma’s radioactivity (36) on the parental representations of the child. They face difficulties to be identified as same by their biological family and their birth culture as well as by their adoptive family and their new host culture.

The uncanny (37) generates dread within the parents psyche and triggers defense mechanisms. Negotiating the dread can result into distancing oneself and rejecting the source of the dread, that is the traumatic residue lodged in the child. This process is echoed in the poor reflective function. Some parents attribute their children’s traumatic experiences to fantasized characteristics of the country of birth, which could be interpreted as an attempt to give their children’s personal experience meaning, to give it a place. The difficulty facing trauma results in disregarding cultural interpretations and resorting to generalizations in order to make sense of an utterly painful situation. It also provides a protective distance with the person’s culture of origin (26). The parents’ accounts show how they are confronted with trauma that cannot be remembered/elaborated. The culture of origin allows them to fill the void of representation. It is the culture of origin, which is foreign and strange, that explains the violence experienced by the children. The trauma is attributed to the culture of origin. Thus, parents are confronted with a double-layered otherness—the otherness of the trauma and the otherness of the culture of birth. This undermines their ability to identify and empathize with their child, who is kept at a distance because of this overwhelming otherness.

Some accounts were characterized by a disorganization, even fragmentation of the narrative, which was contradictory, incoherent, pervaded by a single theme and punctuated by silences or physical symptoms, such as laughing or coughing. The use of stop-gap words and excessive focus on scenes of the past complete the description of the narrative structure. We consider that the parents thus exhibited traces of unresolved parental trauma, as described in the Adult Attachment Interview (27). Hess et al. (38) found evidence of narrative disorganization in their study on the structure of narratives concerning loss or trauma using the Adult Attachment Interview. These findings show a non-metabolization of traumatic events. Disorganization and incoherence in the narrative are only present when the traumatic events are mentioned: the same respondent can give a perfectly coherent account when mentioning other aspects of the history (39).

The present results, showing the traumatogenic impact of children’s pre-adoptive traumatic experience on their parents and on the parents’ representations of their children, can be interpreted on the basis of concepts developed by Wilson et al. (40). These authors suggested two types of counter-transference reactions to a traumatic narrative. Although this model was developed from a therapist’s reaction to his patient’s narratives, it seems interesting in the exploration of parents’ reactions to what is transferred to them—by the children themselves or by others—of their child’s pre-adoptive experience. The first type of counter-transference is characterized by avoidance and the second by over-identification. Our results point to an over-representation of the first type in the interviews. The empathic strain experienced triggers empathic repression (40). These types of reactions to a traumatic account include forms of denial, trivialization, distortion, avoidance, detachment, and withdrawal toward the subject, and these are reactions that were highlighted in our interviews with the parents.

The themes that emerged from the interview analyses showed some evidence of transmission of trauma among adopting parents confronted with their child’s pre-adoptive traumatic experiences. This finding is confirmed by other studies carried out on the subject, underlining traumatic effects among parents as a consequence of their confrontation with their children’s trauma (19) and symptoms of depression, stress, or anxiety they may develop (20).

The analyses presented are, however, limited by the small number of fathers interviewed, in comparison to the mothers. A second limitation, frequent in qualitative research, concerns the difference between parents who volunteered in this study and those who declined. Maybe our participants encounter more difficulties in their parenting process in relation to their child’s pre-adoptive past and volunteered in our research in order to gain understanding of their internal processes.

The present results underline the need for specific therapeutic support for adopting parents, and even more the need for a support program for these parents in their adoption procedure. This support would enable better parental preparation to welcome a child who has lived through pre-adoptive traumatic events. This is all the more true because children are being adopted at increasingly later ages, thus increasing their risk of previous confrontation with hostile environments or traumatic life events. In a previous study, we showed an increased risk for traumatic experiences for parents, when they first encounter their child (41). A pre-adoption program addressed to the parents could make them aware of the reactions and needs of traumatized children, and also of the challenges faced by those who take care of them, in particular the families. Some authors (42–44) have highlighted the need for parental preparation, in order to prepare them to be parents and also therapists for their child. Many studies have in the last few years underlined the usefulness of specific training on trauma for parents—both for welcoming the child (15, 43, 45–47) and for coping with the risk of trauma transmission (16, 19, 20, 48).

## Data Availability Statement

The datasets generated for this study are available on request to the corresponding author.

## Ethics Statement

This study, involving human participants, was reviewed and approved by the Comite´ d’Evaluation de l’Ethique des projets de Recherche Biome´dicale (CEERB) du Groupe Hospitalo-universitaire Nord, on the 29th of March, 2011 (Institutional Review Board N° IRB00006477). The patients/participants provided their written informed consent to participate in this study.

## Author Contributions

SS and AH conducted the research under the supervision of MH. The order of appearance on the manuscript: SS, AH, MH.

## Conflict of Interest

The authors declare that the research was conducted in the absence of any commercial or financial relationships that could be construed as a potential conflict of interest.
